# Structural characterization of a novel KH-domain containing plant chloroplast endonuclease

**DOI:** 10.1038/s41598-018-31142-w

**Published:** 2018-09-13

**Authors:** Ashok K. Rout, Himanshu Singh, Sunita Patel, Vandana Raghvan, Saurabh Gautam, R. Minda, Basuthkar J. Rao, Kandala V. R. Chary

**Affiliations:** 1 Department of Chemical Sciences, Tata Institute of Fundamental Research, Mumbai, 400005 India; 2 Department of Chemical and Biological Sciences, Tata Institute of Fundamental Research, Mumbai, 400005 India; 3 Indian Institutes of Science Education and Research, Berhampur, 760010 Odisha India; 4 Tata Institute of Fundamental Research, Center for Interdisciplinary Sciences, Hyderabad, 500075 India; 5 UM-DAE Centre for Excellence in Basic Sciences, Mumbai University Campus, Mumbai, India; 6 Department Chemistry and Pharmacy, Ludwig-Maximilians-University, Butenandtstr. 5-13, 81377 Munich, Germany; 7 Department of Chemistry, Indian Institute of Technology Delhi, Hauz Khas, New Delhi 110093 India; 8 Indian Institutes of Science Education and Research, Tirupati, 517501 Tirupati, India

## Abstract

*Chlamydomonas reinhardtii* is a single celled alga that undergoes apoptosis in response to UV-C irradiation. UVI31+, a novel UV-inducible DNA endonuclease in *C*. *reinhardtii*, which normally localizes near cell wall and pyrenoid regions, gets redistributed into punctate foci within the whole chloroplast, away from the pyrenoid, upon UV-stress. Solution NMR structure of the first putative UV inducible endonuclease UVI31+ revealed an α_1_–β_1_–β_2_–α_2_–α_3_–β_3_ fold similar to BolA and type II KH-domain ubiquitous protein families. Three α−helices of UVI31+ constitute one side of the protein surface, which are packed to the other side, made of three-stranded β–sheet, with intervening hydrophobic residues. A twenty-three residues long polypeptide stretch (D54-H76) connecting β_1_ and β_2_ strands is found to be highly flexible. Interestingly, UVI31+ recognizes the DNA primarily through its β–sheet. We propose that the catalytic triad residues involving Ser114, His95 and Thr116 facilitate DNA endonuclease activity of UVI31+. Further, decreased endonuclease activity of the S114A mutant is consistent with the direct participation of Ser114 in the catalysis. This study provides the first structural description of a plant chloroplast endonuclease that is regulated by UV-stress response.

## Introduction

*Chlamydomonas reinhardtii* is a single celled alga that swims with its two flagella and undergoes apoptosis in response to UV-C irradiation^1^. The alga shows classical hallmarks of animal cell apoptosis and hence can be used as a model system for studying its molecular mechanism in a plant-like environment. Several candidate molecules such as apoptosis protease activating factor (APAF), a caspase-3 like protein, and a defender against apoptotic death (*DAD1*) were studied in *C*. *reinhardtii*, and found to exhibit a distinct activation pattern correlating with the onset of death upon UV irradiation^1,2^. One of these candidate molecules, which is yet to be characterized, is referred to as UVI31+ (encoded by *uvi31*+ gene, which was originally identified in *Schizosaccharomyces pombe* as an UV-inducible gene)^3,4^. The *uvi31*+ gene is induced by UV light and its expression remains unaltered by other DNA damaging or cytotoxic agents^3^. However, *uvi31*+ does not show any significant homology with any known DNA repair genes^4^. Its expression has been found to be cell-cycle and growth-phase dependent^3–5^. The cellular and biochemical characterization of UVI31+ protein revealed interesting biological facets: UVI31+ in *C*. *reinhardtii* exhibits DNA endonuclease activity and it is induced upon UV stress^3,6^. We have previously observed that UVI31+ is induced in *C*. *reinhardtii* when grown in the dark, a common stressor, whereby the protein localization is enhanced near the pyrenoid^6^. UVI31+ localized near the cell wall and pyrenoid regions gets redistributed into punctate foci within the whole chloroplast, away from the pyrenoid, upon UV stress^6^. The observed induction upon UV-stress and the endonuclease activity suggested a plausible role of UVI31+ in DNA repair^3,6^ or some aspects of cellular adaptations.

The study of DNA repair in algae and in higher plants has been slow in comparison with other eukaryotic organisms including yeast. Interestingly, *C*. *reinhardtii* is routinely used as a model system to study the DNA repair processes, primarily due to the availability of several mutants deficient in various repair processes and their ease of induction and isolation^7^. The most characterized DNA repair process in *C*. *reinhardtii* is the photo-reactivation repair that repairs thymine dimers in DNA^8^. UV irradiation causes the formation of thymine dimers in DNA, which are repaired in plants through two major mechanisms: photo-reactivation repair and excision repair. The participation of DNA photolyase in repair process is reported in *C*. *reinhardtii*^7–9^. DNA endonuclease activity was detected in UVI31+ from *C*. *reinhardtii*, both endogenously and in its purified form^6^. The UVI31+ from *C*. *reinhardtii*, when heterologously expressed in *E*. *coli*, confers to the bacteria around 1000 folds higher resistance to UV^6^. The observed UVI31+ induction upon UV-stress, increased UV resistance upon overexpression in *E*. *coli*, and the localization near pyrenoid and chloroplast regions suggest a plausible role of UVI31+ in DNA repair^6,9^.

Given its dynamic cellular localization changes as a function of various stress treatments (UV and dark incubation) and associated endonuclease activity, seeking the structural biology of UVI31+ as an interesting plant protein became highly imminent. Therefore, in the current study, we sought the basic structural biology of UVI31+ from *C*. *reinhardtii* as a target in order to glean the structural and functional insights of this protein. Moreover, we surmised that the study would enhance our understanding of DNA repair system in plants. Here, we set out to determine the 3D structure of UVI31+ using multi-dimensional solution NMR spectroscopy and study its functional aspects through various biophysical methods and biochemical assays. The study thus provides the structural basis for DNA recognition and endonuclease catalysis by a plant protein UVI31+.

## Results and Discussion

### Primary structure of UVI31+ reveals short stretches of intrinsically disordered regions

The UVI31+ primary structure revealed three short stretches of intrinsically disordered regions (M24-P31, H59-A73 and T120-Q123) as predicted by *CASP9* and *Metadisorder* web servers (Fig. S1)^10^. Rest of the primary structure displayed higher propensity for ordered structure. The primary structure further shows 24% sequence identity and 40.8% sequence similarity with a protein of BolA (*pdb id: 2DHM*) family (Scheme S1 and S2)^11^.

### UVI31+ exists as a stable monomer

The UVI31+ was purified using affinity chromatography followed by size-exclusion chromatography and analyzed by SDS-PAGE, mass spectroscopy (MALDI-TOF) and dynamic light scattering (DLS) experiments. During the size-exclusion chromatography, UVI31+ was eluted at a molecular weight corresponding to its monomeric state (Fig. S2A). UVI31+ showed up as a single-band in SDS-PAGE (Fig. S2B), an indication of its purity and exhibited robust DNA endonuclease activity as assessed through “in-gel” assays^6^. Further, the MALDI data showed monomeric state of UVI31+, with a molecular weight (M^r^) of 13429.85 Da w.r.t. the expected value of 13300.93 Da (Fig. S2C). The DLS measurements confirmed the monomeric state of the protein with 2.31 nm hydrodynamic radius (R_h_) (Fig. S2D). The CD spectrum of UVI31+ suggested that the protein is well-folded (Fig. S2E), while the temperature dependent far-UV CD spectra of the protein revealed its stability with a T_m_ value of ~54 °C (Fig. S2F).

### 3D structure of UVI31+

The 3D structure of UVI31+ was determined by simulated annealing procedure with the torsion angle dynamics protocol using the NMR constraints mentioned in Methods section and the program CYANA 3.0^12,13^. A total of 100 conformers were calculated, from which ten conformers with lowest target function and no distance or angle violations were selected. These ten conformers with lowest target function were further subjected to molecular dynamics simulation in explicit water with NMR-derived distance restraints, angle restraints using CNS 1.21 program^14^ and the standard water shell refinement protocol was used^15,16^. This step improved the Ramachandran plot statistics and also the Z-score for the ordered residues. The Ramachandran plot of the superimposed ensemble showed that 95.2% of residues form part of the most favorable regions of torsion angle space, while 4.7% of the residues were found to be in allowed regions and 0.1% were found in additionally allowed regions. The program PROCHECK-NMR^17^ and PSVS-1.4 (http://www.psvs-1_4.nesg.org) were used to validate the quality of the selected ensemble of lowest energy structures of UVI31+. The 3D coordinates of individual atoms of UVI31+ thus obtained were deposited in the PDB (*pdb id: 5ZB6*).

**Figure 1:**
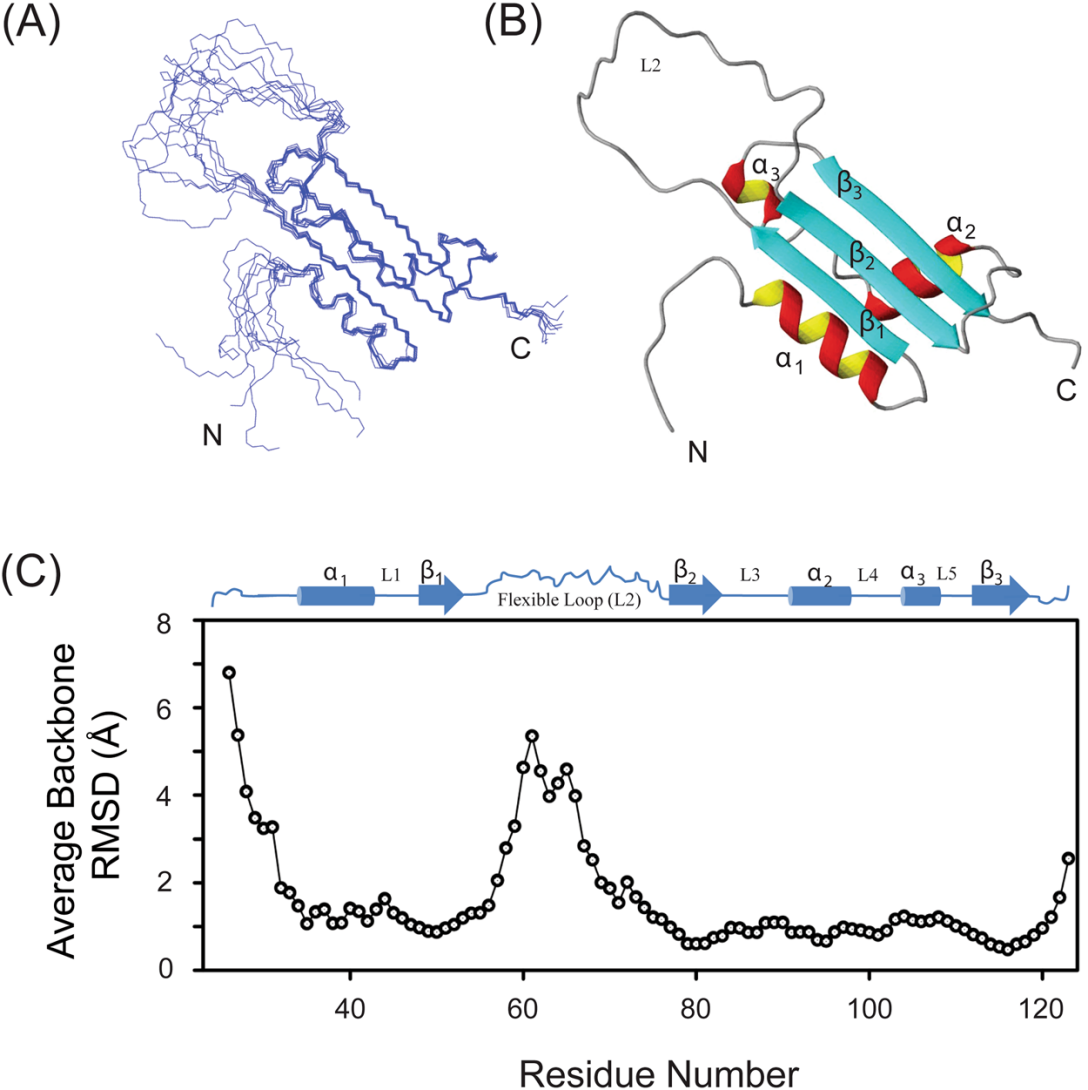
Solution NMR structure of UVI31+. (**A**) An ensemble of 10 superimposed minimum energy NMR-derived structures of UVI31+. (**B**) Representative lowest energy structure of UVI31+. (**C**) Residue-wise plot of RMSD for UVI31+ with NMR derived secondary structural elements shown at the top of this panel.

A superposition of ten lowest-energy conformers and a representative lowest energy structure of UVI31+ with an all backbone RMSD of 0.4 Å (ordered region) are shown in Fig. 1A,B, respectively. The 3D structural statistics are provided in Table 1. The overall structural organization observed in the 3D structure of UVI31+ consisted of three α−helices (α_1_: G34-A43 (with an RMSD = 0.12 Å), α_2_: L91-I98 (0.08 Å), α_3_: E105-A108 (0.16 Å)), three β-strands (β_1_: H48-N53 (with an RMSD = 0.14 Å), β_2_: F77-S83 (0.13 Å), β_3_: A112-K118 (0.11 Å)) and a twenty-three residues long flexible loop (D54-H76). Thus, UVI31+ adopts an α_1_–β_1_–β_2_–α_2_–α_3_–β_4_ fold, with a 23 amino acid residues long highly flexible loop (D54-H76) between the β–strands β_1_ and β_2_. Figure 1C shows residue-wise average backbone RMSD (in Å) derived from the mean structure of the ten energy-minimized conformations of UVI31+, which signify greater flexibility for the polypeptide stretch connecting β_1_ and β_2_ strands.

**Table 1:** NMR structural statistics for the ensemble of 10 refined conformers of UVI31+ (*pdb id:5ZB6*).

Conformationally restricting restraints	
**Distance Restraint List**	
Total	1254
Intraresidue (i = j)	298
Sequential ([i − j] = 1)	646
medium-range	204
long-range	106
Hydrogen bonds	42
Dihedral angle restrains (ϕ and φ)	102
Disulfide restraints	0
No. of restraints per residue	12.66
**Residual restraint violations**	
Average no. of distance violations per structure > 0.5 Å	0
Average no. of dihedral angle violations per structure > 5°	0
Average no. of Van der Waals violations per structure > 0.5 Å	0
**Model quality**	
Rmsd backbone atoms (Å)	0.4
Rmsd heavy atoms (Å)	0.9
Rmsd bond lengths (Å)	0.004
Rmsd bond angles (°)	0.7
**MolProbity Ramachandran statistics** ^**a**^	
Most favored region (%)	95.2
Allowed region (%)	4.7
Additionally allowed region (%)	0.1
Disallowed region (%)	0.0
**Global quality scores (raw/Z score)**	
Verify3D	0.15/−4.98
PROCHECK (ϕ-ψ)	−0.11/−0.12
PROCHECK (all)	−0.66/−3.90
MolProbity clash score	27.60/−3.21
**Model contents**	
Total no. of residues	100
BMRB accession number	16864
PDB ID code	5ZB6

^a^Ordered residues: 33–53, 76–122.

### UVI31+ is similar to the members of BolA and K Homology domain protein families

**Figure 2:**
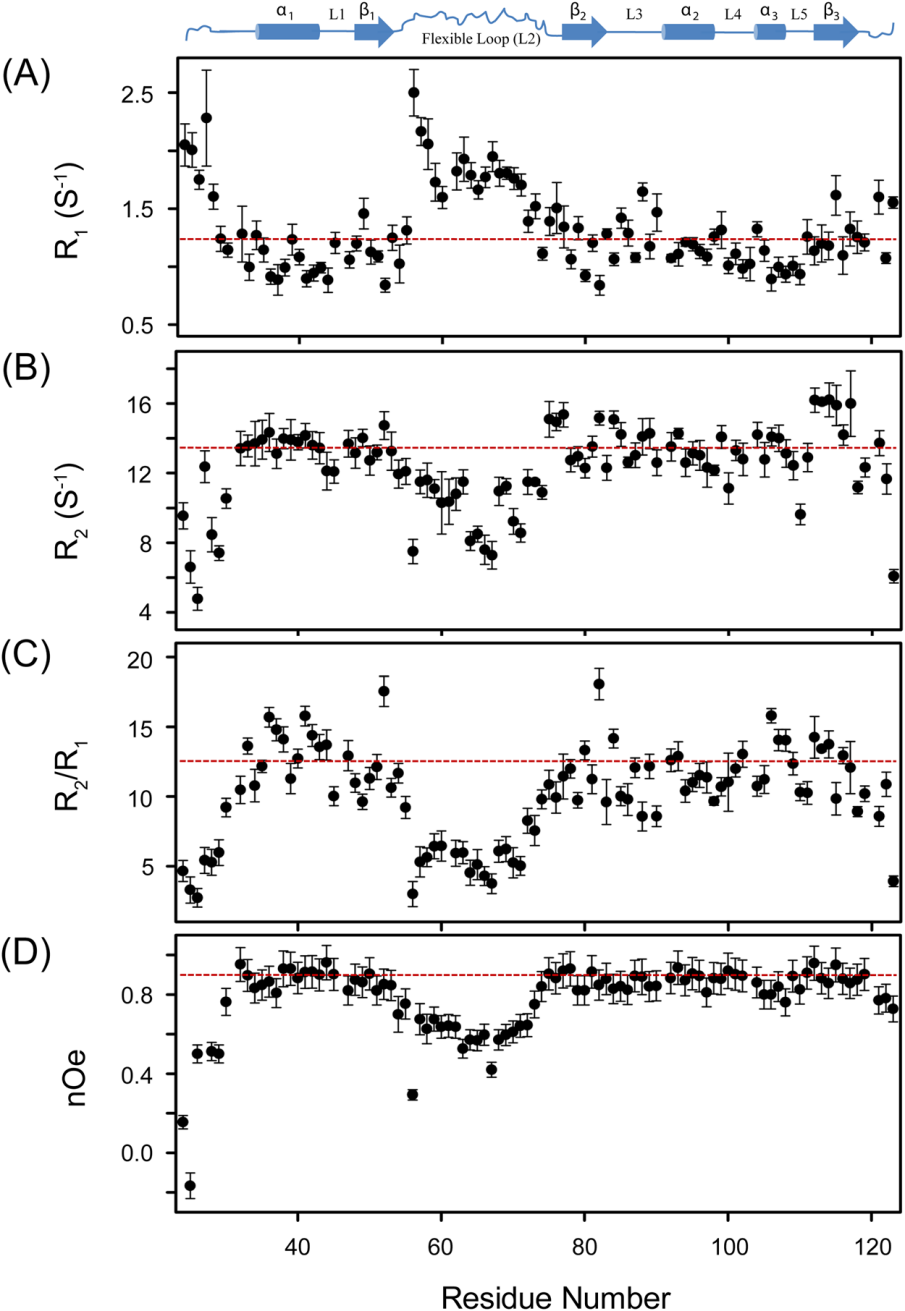
The ^15^N relaxation data of UVI31+ recorded on a Bruker Avance 800 MHz NMR spectrometer at 25 °C. (**A**) Longitudinal relaxation rates, R_1_ (1/T_1_), (**B**) Transverse relaxation rates, R_2_ (1/T_2_), (**C**) Residue-wise plot of R_2_/R_1_, (**D**) [^15^N, ^1^H]-nOe defined as I_sat_/I_eq_, where I_sat_ and I_eq_ are the measured intensities of peaks in the 2D experiments with and without proton saturation, respectively. The relaxation data could not be estimated for Pro residues (P31, P46, and P120) and for residues H61, L91, and S103 due to spectral overlap or weak intensities resulting improper fit. NMR derived secondary structural elements of UVI31+ are shown at the top of the panel A.

*C*. *reinhardtii* UVI31+ shows substantial structural homology with BolA protein from *E*. *coli* with both of them adopting α_1_–β_1_–β_2_–α_2_–α_3_–β_3_ structural fold (Fig. S3A)^11^. A structure comparison using DALI webserver (http://ekhidna.biocenter.helsinki.fi/dali_lite/start) results a Z-score of 3.3. The structured parts of the two proteins are superimposed with an RMSD of 3.8 Å. *E*. *coli BolA* and its homologues constitute a widely-conserved BolA-like protein family and they have divergent functions^18–21^. These families of proteins have the ability to impart round cell morphology when over-expressed in bacterial cells following a stress response^22^. In addition, BolA family proteins exert cell division septation control and also show DNA binding ability^23^. BolA is found in prokaryotes and eukaryotes including *Homo sapiens*. On the basis of functional genomics and 3D structural data, BolA was predicted to be a reductase that interacts with a mono-thiol glutaredoxin^24^. Recently, researchers have found that deletion of *BolA* in *Plasmodium falciparum* resulted in its slow growth, morphological defects and accumulation of high levels of reactive oxygen species^25^. Thus, BolA-like proteins are considered as potential precursors to new anti-malarial drugs in therapeutics. This highlights the importance of this family of proteins and their fold. Further, BolA proteins have a helix-turn-helix motif, which is a major structural motif with an ability to bind DNA, supporting the postulated function of DNA repair^11,26^. However, the molecular function of BolA remains unknown till date^19^. It is worth to mention here that while the BolA protein has 16 residues long-loop between β_1_ and β_2_ strands, UVI31+ has a 23 residues long-loop (D54-H76) between β_1_ and β_2_ strands (Fig. S3A). This loop is poorly defined, primarily due to insufficient nOes seen within this polypeptide stretch, a consequence of greater flexibility. The flexibility of this loop is further confirmed from the ^15^N-relaxation data and [^15^N, ^1^H]-nOe data, as discussed below (Fig. 2). These flexible long-loops of UVI31+ and BolA (*pdb id: 2DHM*) could be superimposed poorly with an RMSD of 2.3 Å and the loop in UVI31+ was found to adopt a more open conformation as compared to that in BolA^11^.

Another structural homologue of UVI31+ is found to be the K Homology (KH) domain, which also adopts α_1_–β_1_–β_2_–α_2_–α_3_–β_3_ structural fold (Fig. S3B)^27–29^. A structure comparison using DALI webserver results a Z-score of 2.3 and have an RMSD of 4.5 Å for the structured region. This KH domain was first identified in the human heterogeneous nuclear ribonucleoprotein (hnRNP) K^30^. An evolutionarily conserved sequence of around 70 amino acid residues, the KH domain is present in a wide variety of nucleic acid-binding proteins associated with transcriptional and translational regulation, along with other cellular processes^27–29,31^. KH domains bind either RNA or single stranded DNA^11,32–34^. The nucleic acid is bound in an extended conformation across one-side of the domain with nonspecific contacts, contributing to its binding specificity. There are two types of KH domains. The type I KH domain has a three-stranded β–sheet with its β–strands in anti-parallel orientation with respect to one another. On the other hand, in the type II KH domains, two of the β–strands belonging to the three-stranded β–sheet are in a parallel orientation^11,33,35^. Besides, it has been highlighted that β–β–α metal topology seen in KH domains acts as prevalent nuclease domain in many nucleases^36–40^.

The orientation of all α–helices and β–strands present in UVI31+ is almost similar to the arrangement in BolA and K-homology type II domain proteins (Fig. S3A,B). While β_1_ and β_2_ strands are in an anti-parallel orientation, β_2_ and β_3_ strands are found to be in parallel orientation with respect to each other (Fig. 1B). The long loop connecting β_1_ and β_2_ is missing in both BolA and KH domain. The orientation of the α–helices (α_1_, α_2_ and α_3_) in the UVI31+ is similar to that of BolA and K-homology domain protein family^11,28^. In UVI31+, the three α–helices form one side of the protein surface, while the other side is formed by three β–strands and a strong hydrophobic core packed between them, providing a compact 3D structure (Fig. 1A,B). Most of the hydrophobic residues (Ala, Leu, lle, Val, Pro, Phe, Trp and Met) form the central core of the protein and are buried at the interface of α–helices and β–sheet. The highly flexible loop (D54-H76) between β_1_ and β_2_ strands is devoid of branched hydrophobic residues and it has only four Ala residues spaced widely apart suggesting its intrinsically disordered nature.

### Modulation of conformational dynamics of UVI31+

The ^15^N relaxation data aid in probing milliseconds to picoseconds motions and provide information about the overall and internal motions in a given protein and thus are very crucial for understanding the protein dynamics^41^. The ^15^N-longitudinal relaxation rates (R_1_) and [^15^N, ^1^H]-nOe values provide information about fast time-scale dynamics in the range of nanoseconds to picoseconds time-scale. On the other hand, the ^15^N-transverse relaxation rates (R_2_) are largely sensitive to slow motions and reflect slower exchange processes that occur in the milliseconds to microseconds time scale^42^. The R_1_ and R_2_ values determined for individual residues present in the UVI31+ show large variations all along the sequence (Fig. 2A,B). The average R_1_ and R_2_ values for the residues involved in highly structured regions were found to be 1.14 ± 0.17 s^−1^ and 13.77 ± 1.20 s^−1^, respectively, while for the loop connecting β_1_ and β_2_ strands these values were 1.69 ± 0.33 s^−1^ and 10.69 ± 2.39 s^−1^, respectively. The relative higher R_1_ values and lower R_2_ values for the loop connecting β_1_ and β_2_ strands indicate that the loop is highly flexible, as discussed. This observation is further supported by a low average R_2_/R_1_ value of 6.85 ± 2.46 for the loop as compared to that of the structured regions, which was found to be 12.35 ± 2.04 (Fig. 2C). The flexibility of the polypeptide stretch D54-H76 was also evident from the observation of a low average value for the [^15^N, ^1^H]-nOe, which was found to be 0.64 ± 0.14 as compared to that of the structured regions, which was found to be 0.87 ± 0.05 (Fig. 2D). In addition, the negative and low nOe values seen near the N- and C-terminals ends of UVI31+ indicate their flexibility too. Taken together, both the NMR derived 3D structure and ^15^N-relaxation data of UVI31+ clearly suggest that the protein is quite ordered in entirety except for the terminal ends and the polypeptide stretch (D54-H76) connecting β_1_ and β_2_ strands present in the protein.

### Electrostatic surface charge potential distribution in UVI31+

**Figure 3:**
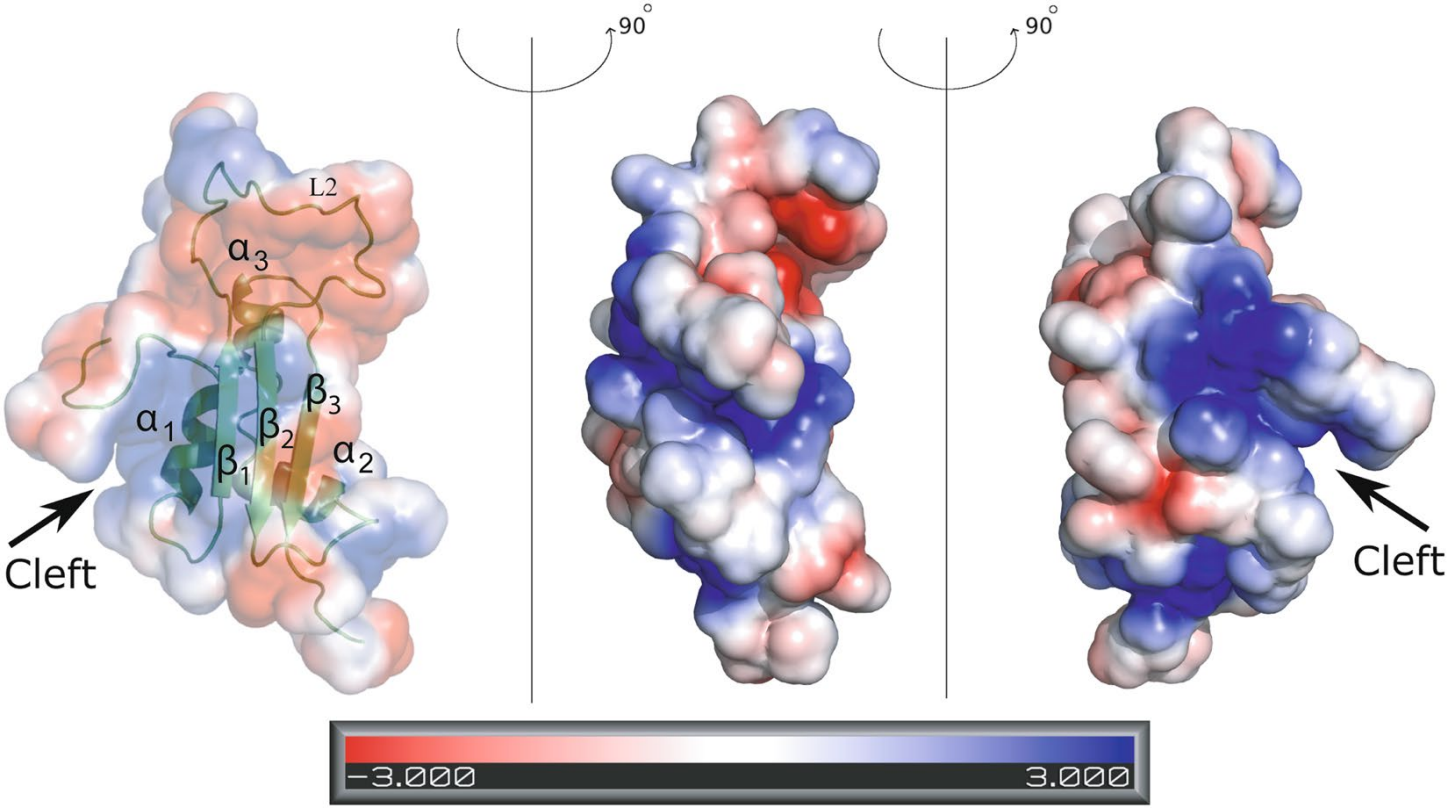
Surface electrostatic charge potential of UVI31+. The spectral bar at the bottom indicates the electrostatic potential in electron volts (eV). Red color represents negative charge potential (−3 eV) while blue color represents positive charge potential (3 eV).

In order to map potential DNA binding surface of the protein, we calculated the electrostatic surface charge potential for UVI31+. As shown in the Fig. 3, the highly flexible disordered loop (D54-H76) showed significant amount of negatively charged surface potential (shown in red). On the other hand, we observed pronounced positively charged surface potential (in blue) right at the center of the protein, forming a distinct cleft (Fig. 3). The residues in and around this cleft belong to the N-terminal segment (M24-A35), proximal α_1_ helix and β_1_ strand (H48-N53). The residues that significantly contribute to the positively charged cleft are K37, K39, K50, K57, R78 and K107. This cleft is the most plausible binding site for the negatively charged DNA/RNA, facilitating the endonuclease activity of the protein, which we probed further.

### Residues belonging to the β–sheet and the long loop regions undergo perturbation upon DNA binding

**Figure 4:**
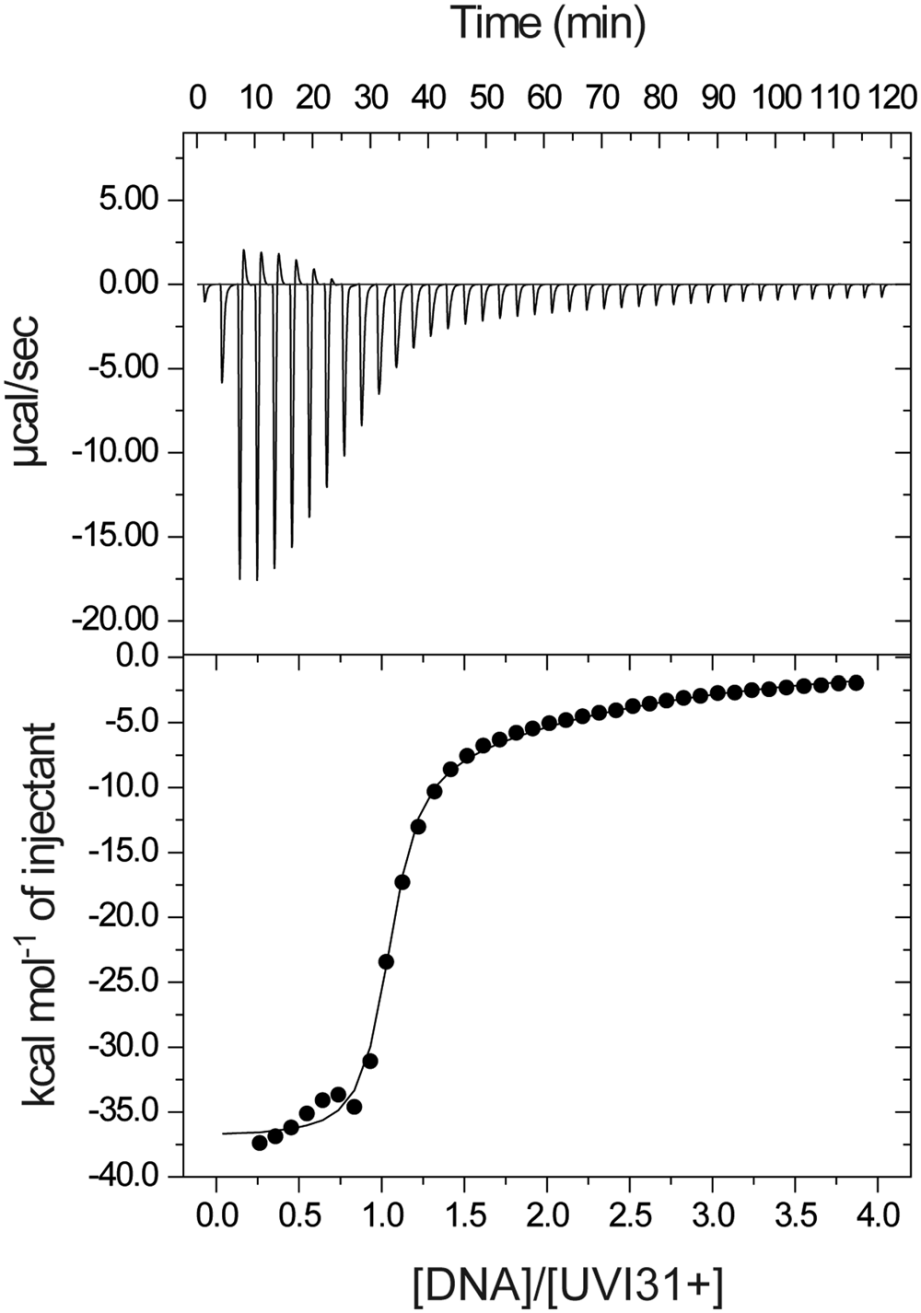
ITC data of the binding of UVI31+ to ds-DNA. The thermodynamic parameters describing the fit are presented in Table S1.

To test the DNA binding affinity of UVI31+, ITC experiments were performed with a self-complementary ds-(CGCGAATTCGCG) as a template to measure apparent dissociation constant (K_d_) (Fig. 4). The ITC isotherms showed that UVI31+ binds to ds-DNA exothermically (Fig. 4 and Table S1), with effective K_d_ value of 5.36 μM. The binding occurs at two sets of sites (Table S1).

**Figure 5:**
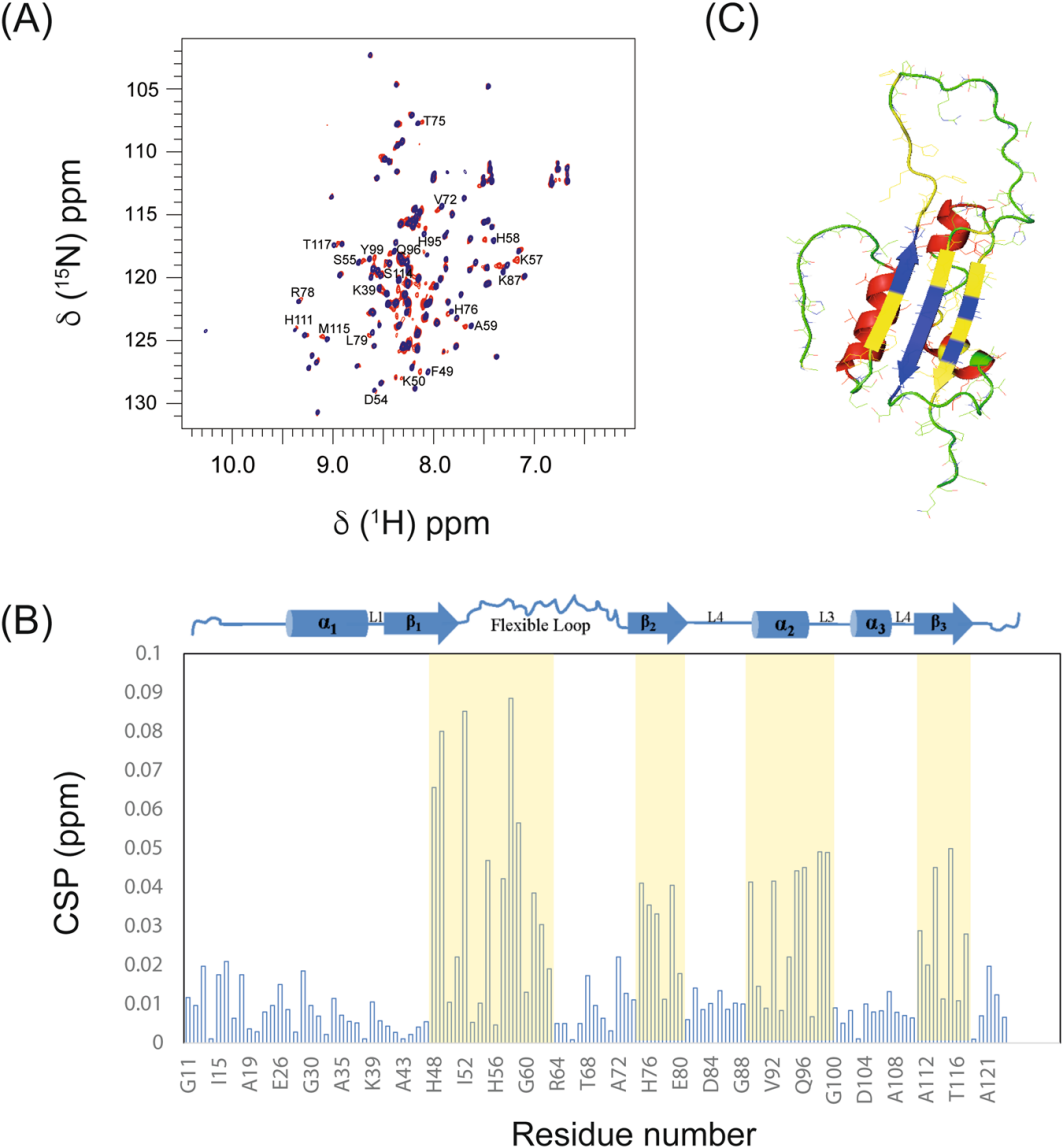
UVI31+ and DNA interaction by NMR. (**A**) Overlay of 2D [^15^N, ^1^H]-so-fast-HMQC for UVI31+ (black) and UVI31+: DNA complex (red). (**B**) Residue-wise plot of CSPs upon UVI31+: DNA complex formation. Light yellow bar represents residues involved in DNA interaction. The secondary structural elements of the NMR derived structure of UVI31+ are depicted on top of the panel B. (**C**) The α-helices, β-strands and loops are shown in red, blue and green, respectively. The residues undergoing perturbation upon DNA binding are shown in yellow.

The gel-filtration chromatography of the purified UVI31+ protein showed that its biochemical endonuclease activity was associated with monomeric form of the protein, as was reported earlier^6^. However, the identity of the residues participating in the DNA recognition was not known. Nuclease activity of UVI31+ with DNA fragments of varying lengths showed that the protein binds strongly to 12-bp DNA and the binding was not detectable when DNA size was less than 10-bp and as discussed below, UVI31+ has no strong target preference for either ssDNA or dsDNA (Fig. S4). With this in the backdrop, the 12 mer ds-DNA mentioned above was chosen for interaction studies with UVI31+. In the present study, we could identify these residues by recording a 2D [^15^N, ^1^H]-so-fast-HMQC, with an acquisition time of 2 s. An overlay of [^15^N, ^1^H]-so-fast-HMQC spectra of UVI31+ and UVI31+:DNA complex (Fig. 5A) showed up significant CSPs indicating the interaction of UVI31+ with the ds-DNA. Residue-wise CSPs calculated as described in the Methods section enabled us to identify the interacting residues of UVI31+ with ds-DNA. As is evident from the CSP plot (Fig. 5B), residues belonging to β_1_ (F49 and K50), β_2_ (L79), β_3_ (K118 and T119) strands, four residues (K57, H58, A59 and H76) of the flexible long-loop (D54-H76) between β_1_ and β_2_ strands and two residues (A35 and K37) belonging to the α_1_ helix undergo significant CSPs upon DNA binding (Fig. 5C). We highlight here that out of these eleven residues, six are positively charged. Three of these residues K37, K50 and K57 contribute significantly to the total positive surface-charge potential and form part of the cleft mentioned earlier (Fig. 3). These residues interact with the negatively charged DNA. As discussed earlier, though the protein possesses three α−helices, only two residues belonging to the α_1_-helix were found interacting with DNA, suggesting no involvement of α_2_- and α_3_-helices in DNA recognition. Other residues, which showed CSPs, are those belonging to the flexible loop (K57, H58, A59 and H76). Topologically, these residues are situated in close proximity to the β–sheet (Fig. 5C). The K35 belonging to the α_1_-helix is in close proximity to K50 of β_1_ strand and L79 of β_2_-strand with the corresponding C^α^-C^α^ distances 11.0 and 7.6 Å, respectively. The residues belonging to α_1_-helix, which show chemical shift perturbations, are in close proximity to the flexible loop. All these observations taken together suggest that the DNA recognition is with the loop and β–sheet domain of UVI31+.

Taking into account the fact that few residues belonging to the flexible long-loop (D54-H76) undergo CSPs upon interaction with the DNA, we attempted to study the influence of the flexible long-loop on the endonuclease activity. In this endeavor, shortening of the 23 residues long-loop connecting β_1_ and β_2_ strands to a 4 residues loop (^54^DSGG^57^) (Fig. S5A), as described in the Methods section, did not affect the endonuclease activity. The zymogram convincingly showed that the DNA endonuclease activity of “loop-null” mutant is comparable to that of the wild-type UVI31+ (Fig. S5B).

### S114A mutation drastically reduced endonuclease activity of UVI31+

With the 3D structure in hand and using the computational method called CLASP (CataLytic Active Site Prediction), we attempted to uncover putative residues, namely Q96, Y99, E105 and S114 that showed good congruent matches for nuclease activity^43^. This is based on the spatial and electrostatic properties of the probable catalytic residues^43^. Out of all these, we speculated that the S114 could be responsible for the endonuclease activity as the Ser hydroxyl group is implicated as a nucleophile in the catalysis of nucleases action^36^.

On the other hand, it is well known fact that an acid-base-nucleophile catalytic triad is a group of three amino-acid residues that are found in and around the active site of several nucleases and some proteases^44^. Such a triad is a common motif involved in generating a nucleophilic residue that is needed for covalent catalysis^44^. The side-chain of the nucleophilic residue performs covalent catalysis of the substrate. The lone-pair of electrons present on the oxygen or sulphur attacks the electropositive carbonyl/phosphoryl group. The “Ser-His-Glu/Asp/Thr” motif is one of the most thoroughly characterized catalytic triad^45–49^. As mentioned earlier since the side-chain hydroxyl group of a Ser is implicated as a nucleophile in the catalysis of nucleases action, one could in principle identify a possible catalytic triad by searching around for the partner residues (His and Glu/Asp/Thr) in the vicinity of any Ser present in a given protein. For example, in UV-damage endonuclease (UVDE, *pdb id: 2J6V*) protein, the S234, which was earlier implicated in the endonuclease activity, was considered to be part of a catalytic triad (Ser-His-Glu/Asp/Thr)^50^. This residue was used as a reference in identifying other two partner residues (His-Glu/Asp/Thr) in its vicinity. Such a search revealed H244 and E269 as partner residues. Likewise, in the present study, the putative endonuclease active site in UVI31+ was predicted by identifying a potential catalytic triad (Ser-His-Glu/Asp/Thr) likely to be present in its 3D structure. In UVI31+, there are eight Ser residues. By taking each of these Ser residues as a reference, search was made to identify the putative catalytic triad (Ser-His-Glu/Asp/Thr). This resulted in the prediction of the residues S114, H95 and E80/T116 as the only probable constituents of the catalytic triad (Ser-His-Glu/Thr). A closer examination of the 3D structure revealed that S114, H95 and T116 are in the cleft of UVI31+ as discussed above while E80 is protruding out of the β_3_-strand of the three-stranded β-sheet and exposed to the solvent. Thus, we speculated S114-H95-T116 as the catalytic triad and set out to carry out functional assessment of the promiscuous S114 belonging to the S114-H95-T116 triad by mutating Ser114 to Ala. It is worth to mention here that ‘His’ side-chains play an important role in most of the enzymatic catalytic processes. The pKa of side-chain imidazole proton of free ‘His’ is around 6.2. However, during catalysis it has been observed to cover a pKa range from 6.9 to 7.9. In the case of the catalytic triad involved in DNA binding, ‘His’ pKa value was reported to be 7.3^51–54^. In our proposed catalytic triad, we are expecting ‘His’ pKa value to be in the same range.

CD experiments with this single mutant of UVI31+ (S114A-UVI31+) showed no change in the secondary structure as compared to the wild-type (Figs S6A and S2E). The melting temperature (T_m_) of the mutant and wild-type proteins calculated from the CD data were found to be almost same (~54 °C) (Figs S6B and S2F). Further, the MALDI data showed the mutant is in monomeric state with a molecular weight (M^r^) of 13414.52 Da and the mass is less than 16 Da from the wild-type because of serine to alanine mutation (Figs S6C and S2C). Overlay of 2D [^15^N-^1^H]-so-fast-HMQC of UVI31+ with that of the S114A-UVI31+ showed similar spectral signatures except for the residue mutated, indicating that there are no major structural differences between the wild-type protein and its S114A-UVI31+ (Fig. S6D). These results show that S114A mutant is structurally similar to the wild-type protein.

It is relevant to reiterate here that using a computational method called CLASP (CataLytic Active Site Prediction)^43^, we had pinpointed S114 as a potential catalytically relevant Ser amongst eight available Ser residues in the protein. As predicted, S114A mutation abolished the endonuclease activity, thereby strengthening our prediction. With the 3D structural elucidation (current study), it became feasible to narrow down the putative catalytic triad partners of Ser114. Our structure based search uncovered that S114-H95-T116 could be the best suited triad in UVI31+ that satisfies the geometric constraints of acid-base catalysis requirement which is consistent with CLASP results as well as S114A loss of activity. Therefore, we strongly suggest that S114-H95-T116 is the putative catalytic triad involved in UVI31+ endonuclease activity.

Further, we noticed that the identified catalytic triad (S114-H95-T116) is located in the positively charged cleft, which is shown as blue surface potential in the UVI31+ 3D structure (Fig. 3), hinting its involvement in the interaction with the DNA. There are several reports in literature highlighting the involvement of Ser in hydrogen bonding interactions with the nucleotide bases^55–58^. DNA titrations with S114A mutant showed minimal CSPs, as shown in the overlay of 2D [^15^N, ^1^H]-so-fast-HMQC spectra (Fig. S6E). Overlay of residue-wise CSPs of UVI31+ and S114A-UVI31+ with ds-DNA, showed significantly small CSPs for the residues belonging to β_1_ (F49 and K50), β_2_ (L79), β_3_ (K118 and T119) strands, four residues (K57, H58, A59 and H76) of the flexible long-loop (D54-H76) between β_1_ and β_2_ strands and two residues (A35 and K37) belonging to the α_1_ helix in the case of S114A-UVI31+ mutant protein with ds-DNA (Fig. S6F).

**Figure 6:**
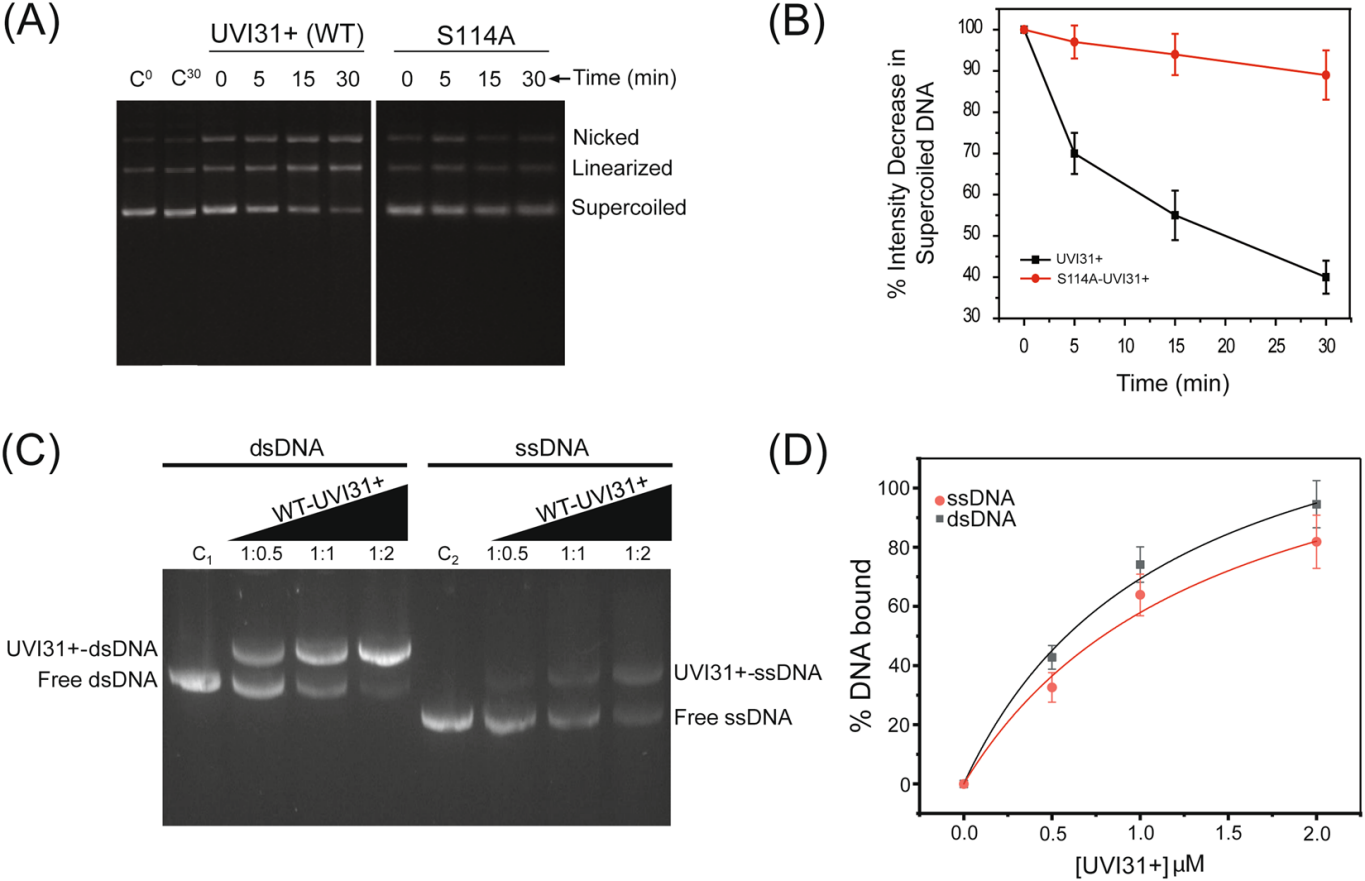
UVI31+ target DNA analyses: ssDNA versus dsDNA. (**A**) UVI31+ endonuclease activity as studied by agarose gel (1%) assay for wild-type UVI31+ and its S114A mutant. The image presented here is from a single agarose gel, cropped and grouped together with white space. The white space represents some of the lanes removed, which contained markers that are not needed for the results presented here. (**B**) Percentage band-intensity decrease of supercoiled DNA plotted as a function of reaction time for wild-type UVI31+ and its S114A mutant. Error bars represent the standard deviation derived from three independent experiments. C° and C^30^ shown in the gel are the plasmids at 0 and 30 min, respectively. (**C**) Electrophoresis mobility shift assay (EMSA) for DNA-UVI31+ interaction studies. Increasing concentration of UVI31+ (0.5 to 2 μM) was used for observing interaction with 50-bp dsDNA or ssDNA using 2% agarose gel electrophoresis. DNA concentration was kept fixed (1.0 μM) in both sets of experiments with increasing UVI31+ protein concentration thereby providing DNA to protein ratios of 1:0.5, 1:1, and 1:2, respectively. C1 and C2 lanes are the control dsDNA and ssDNA bands without protein, respectively. The image presented here is the full-length agarose gel, cropped for presentation point of view. (**D**) Quantification of the gel shift assay was performed via intensity analysis using ImageJ. Results are presented as an average of three measurements and the K_d_ values are presented in Table 2.

Thus, the NMR data suggests that S114A mutant may cause an altered DNA-binding specificity, thereby drastically reducing the endonuclease activity, as seen in DNA endonuclease activity assay (Fig. 6A). It is evident from Fig. 6 that the super-coiled plasmid DNA pBR322 is nicked into its linear and open circular forms by wild-type protein within a time interval of 30 min. In an identical assay with S114A mutant, the nicking activity of the super-coiled plasmid DNA got reduced by ~85% as compared to that of wild-type protein (Fig. 6B). Further, we have performed gel shift assays with ssDNA and dsDNA (Fig. 6C). These gel shift assays clearly demonstrate that UVI31+ binds to both dsDNA and ssDNA. With increasing protein concentration, the intensity of the upper band (complex) got enhanced with concomitant reduction in the intensity of lower band (unbound DNA), demonstrating the formation of protein-DNA complexes with both ligands (dsDNA and ssDNA). Partial loss in the intensity of ssDNA (bound as well as free) as compared to dsDNA could be a result of higher endonuclease activity in the case of ssDNA, which is consistent with our other data (Fig. S4), as discussed in the above paragraphs. We notice that ssDNA versus dsDNA differences may not be very significant: Firstly, the native gel assay might mask internal cleavages in dsDNA and secondly, the ssDNA templates might have residual secondary structures, unless these are homo-polymeric sequences, which in turn present other challenges. Notwithstanding these caveats, direct comparison by gel-shift assay revealed only a minor binding preference of dsDNA over ssDNA. Quantification of the gel shift assay resulted in determining K_d_ values for ssDNA and dsDNA, which turned out to be as 1.4 ± 0.3 μM and 1.1 ± 0.3 μM, respectively (Fig. 6D and Table 2). All these observations put together, point towards UVI31+ protein as DNA endonuclease with no strong target preference for either ssDNA or dsDNA. It is worth mentioning here that the S114-UVI31+ equivalent is missing in BolA (*pdb id: 2DHM*) protein, rendering the protein deficient in endonuclease function although the protein is structurally akin to UVI31+. Interestingly, we did observe partial conservation of catalytic triad in BolA structure (*pdb id:2DHM*) with an A85 in an equivalent structural position of S114 (Fig. S7).

**Table 2:** K_d_ values as determined by gel shift assay (EMSA).

Conditions	K_d_ (μM)	R value
UVI31 + -ssDNA	1.4 ± 0.3	0.98
UVI31 + -dsDNA	1.1 ± 0.3	0.99

## Conclusion

We have determined NMR-derived 3D structure of the UV inducible gene product, UVI31+ protein, from *Chlamydomonas reinhardtii*, which possesses a α_1_–β_1_–β_2_–α_2_–α_3_–β_3_ fold, similar to the fold found in BolA protein family and KH domain type II proteins. The structural elucidation uncovered two important features of the UVI31+ protein structure. Though the domain structure is very similar to that of BolA protein family and KH domain proteins, there were interesting differences vis-à-vis the same with respect to the flexible regions of UVI31+. Further, UVI31+ is found to recognize the DNA primarily by its β-sheet domain. The single point mutation (S114A) showed drastic loss of DNA endonuclease activity in UVI31+, whereas deletion of the long flexible loop had no effect on the endonuclease activity. Based on the 3D structure elucidated in the current study, and the geometric and distance constraints, S114-H95-T116 triad is the putative catalytic triad facilitating the endonuclease activity of UVI31+ protein. Interestingly, the catalytic triad S114-H95-T116 is strategically located in the positively charged cleft of UVI31+ 3D structure. Thus, the 3D structure of UVI31+ reveals a compact core that harbors the nuclease triad residues and a long disordered loop, which imparts the required flexibility to interact with other bio-macromolecular surface such as pyrenoids in the cell. Further, this study reveals the first structural description of a plant chloroplast endonuclease that is regulated by UV-stress response in *C*. *reinhardtii* algal plant. Future studies focused on details of UVI31+ endonuclease mechanism in the context of chloroplast biology will critically hinge on the current results.

## Material and Methods

### Cloning, overexpression, isolation and purification

The cDNA encoding *uvi31+* was cloned into a pET28a expression vector and transformed into *E*. *coli* strain BL21(DE3) cells for over-expression, isolation and purification of the protein as described earlier^59^. Similar procedure was used to clone, overexpress, isolate and purify the S114A mutants of UVI31+^60^. Besides, yet another variant of UVI31+, where in the polypeptide stretch ^54^DSHKHAGHYARDGSTASDAGETH^76^ was replaced with ^54^DSGG^57^ and overexpressed, isolated and purified following the same protocol as that of wild-type protein. All of these mutations were confirmed by DNA sequencing.

### Dynamic light scattering

The hydrodynamic radii (R_h_) of UVI31+ and its mutants were determined using dynamic light scattering (DLS) method on a Dynopro-LS instrument at 830 nm. The protein samples were centrifuged at 13,000 rpm for 10 min and filtered through a syringe filter of 0.45 μm (Millipore) before transferring into a quartz cuvette. The measurements showing parabolic curves with a straight baseline were considered for the estimation of the mean values of R_h_ and associated standard deviations. Regularization plots were used to determine the respective R_h_.

### Circular dichroism (CD) experiments

CD spectra were recorded on a JASCO J-810 spectropolarimeter equipped with a peltier cell temperature controller. For recording the CD spectra in the far-UV region (250–200 nm) 0.1 cm path length quartz cell cuvette was used, while in the visible region (300 to 700 nm) a 1 cm path length quartz cell cuvette was used. The CD parameters chosen were as follows: scan speed of 20 nm/min, time constant of 1.0 s, 1.0 bandwidth, and sensitivity of 100 mdeg. The CD spectra were analyzed using Yang’s method to estimate the secondary structure content of UVI31+ and its mutants. All these experiments were conducted under a flow of pure nitrogen. A good signal-to-noise ratio in the CD spectra in both the spectral ranges were obtained upon data averaging over three scans. The protein concentrations used for far-UV and visible CD studies were of 20 μM.

### Thermal unfolding

The temperature dependence of the visible CD was monitored to address thermal unfolding of UVI31+ and its mutants. The temperature was increased from 20 to 90 °C with a heating rate of 1 °C/min. The protein samples were equilibrated at each temperature for at least 3 min, and the reversibility of the unfolding was ensured by decreasing the temperature with a cooling rate of 1 °C/min. In thermal unfolding experiments, fractions of the unfolded protein f_U_, at different temperatures (T), were calculated as the ratio of (Ɵ_t_ - Ɵ_U_) and (Ɵ_F_ - Ɵ_U_), where, Ɵ_t_ is the observed ellipticity at any temperature, Ɵ_F_ is the ellipticity of the fully folded form and Ɵ_U_ is the ellipticity of the unfolded form.

### NMR spectroscopy

For NMR studies, uniformly ^15^N-labelled (u-^15^N) and ^13^C/^15^N-doubly-labelled (u-^13^C/^15^N) UVI31+ and its mutants were prepared in a mixed solvent of 90% H_2_O and 10% ^2^H_2_O (50 mM sodium phosphate, 50 mM NaCl (pH = 6.4)) as described earlier^59^. All NMR experiments were carried out at 25 °C with protein concentrations between 0.5 and 0.6 mM on a Bruker Avance 800 MHz NMR spectrometer equipped with a 5 mm cryogenically cooled triple-resonance probe and a pulse-field gradient. A suite of 3D double- and triple-resonance NMR experiments were performed for sequence specific ^1^H, ^13^C and ^15^N backbone resonance assignments as discussed earlier^61,62^. In addition, we recorded 3D experiments such as HCCH-COSY/TOCSY, [^15^N, ^1^H]-NOESY-HSQC (τ_m_ = 80 ms) and [^13^C, ^1^H]-NOESY-HSQC (τ_m_ = 120 ms) for almost complete assignment of ^1^H, ^13^C and ^15^N side-chain resonances and for the measurement of nOes used in the 3D structural calculation of the protein. The near complete ^1^H, ^13^C and ^15^N resonance assignments of UVI31+ and its variants were deposited earlier in BMRB (http://www.bmrb.wisc.edu; under the accession numbers 16864 (for wild-type) and 18567 (S114A mutant)^59,60^. The backbone ^15^N T_1_ relaxation measurements at 800 MHz were acquired using 256*1024 complex points along t_1_ and t_2_ dimensions, respectively, and inversion recovery delays of 50, 100, 200*, 300, 400, 500, 600*, 800 and 1020 ms. The ^15^N T_2_ measurements were carried out with the same acquisition parameters using CPMG pulse sequence with relaxation delays of 10, 30, 50*, 70, 90, 110*, 130, 150, 170 and 190 ms. In both the experiments, delays marked with an asterisk were recorded twice for error calculation. Steady-state [^15^N, ^1^H] heteronuclear nOe measurements were carried out with and without proton saturation during the relaxation delay. In these nOe experiments, a 2.5 s of proton saturation was used. The heteronuclear nOe values were determined as the ratio of the peak intensities measured from the spectra acquired with and without proton saturation. NMR spectra were processed using Felix 2002 (*Accelrys Inc*., *San Diego*) and analyzed using Topspin 2.0 and 3.1 (Bruker BioSpin: http://www.bruker-biospin.com/), TATAPRO^63^ and CARA^64^. The ^1^H chemical shifts were referenced with respect to an external standard 2, 2-dimethyl-2-silapentene-5-sulfonates (DSS), while the ^13^C and ^15^N chemical shifts were referenced indirectly^65^.

### NMR structure calculation

The 3D solution structure of UVI31+ was determined using the following NMR constraints: (i) Dihedral angle constraints derived using TALOS with the knowledge of individual ^1^H^N^, ^15^N, ^13^C^α^, ^13^C^β^, ^13^C, chemical shift values as input^66^. A total of 102 ϕ and ψ dihedral angle constraints were used; (ii) Generic hydrogen bond (H-bond) constraints were imposed for residues located at well-defined α-helical and β-strand regions. An upper limit of 2.0 Å was used for H-O distance in all hydrogen bond constraints. Total number of H-bond constraints used was 42; (iii) Cross peaks in NOESY spectra were identified, assigned and the corresponding peak intensities were translated into ^1^H-^1^H distances. A total of 1254 distance constraints, which included 298 intra-residue, 646 inter-residue (sequential), 204 medium-range, and 106 long-range distance constraints were used in the 3D structure calculation. With all these restraints as input, the 3D structure of UVI31+ was calculated using the the program CYANA 3.0^12,13^. The structure figures were prepared using Pymol (The PyMOL Molecular Graphics System, Version 1.8 Schrödinger, LLC) and MOLMOL^67^. The electrostatic surface charge distribution plot was made from the NMR derived structure of UV31+ using APBS tool of PyMOL^68^.

### UVI31+ and DNA interaction by NMR

In order to study the interaction of UVI31+ and DNA, we added 2 µL of 2 mM self-complementary ds 5′-(CGCGAATTCGCG)-3′ DNA (12 mer ds-DNA) with 200 µM (^15^N)-UVI31+ and equilibrated at 25 °C and followed it by recording a series of 2D [^15^N, ^1^H]-so-fast-HMQC spectra at different concentrations of the ds-(CGCGAATTCGCG) DNA^69–71^. The chemical shift perturbations (CSPs) for all the individual residues of UVI31+ were monitored with the knowledge of wild-type UVI31+ ^1^H^N^ and ^15^N resonance assignments^59,60^. The CSPs were measured as [(ΔH)^2^ + (ΔN/10)^2^]^1/2^, where, ΔH and ΔN signify the changes in ^1^H^N^ and ^15^N chemical shifts, respectively. The factor 10 for ^15^N chemical shift was taken as the normalization factor, since the overall range of nitrogen chemical shifts is roughly 10 times that of proton chemical shifts for the backbone amides in folded proteins.

### DNA endonuclease activity assay

The DNA gel assay is a classical way of showing the endonuclease action of a protein on DNA substrate. Conversion of supercoiled DNA to nicked circular DNA followed by linear form of DNA establishes DNA nicking as a function of time^43^. For agarose gel assay, each reaction mixture (20 µl total volume) contained 300 ng of negatively super-coiled pBR322 DNA, taken in 50 mM sodium phosphate (pH 7.6), 50 mM NaCl, 1 mM MgCl_2_ and UVI31+ or its variants. It was followed by incubation of the reaction mixture at 37 °C for 30 min. Reaction was quenched by adding 5 µl stop solution (10% glycerol, 0.005% bromophenol blue, 0.1% SDS). DNA samples were analyzed by gel electrophoresis at 3 V/cm for 4 hr on a 0.8% agarose gel taken in Tris-acetate-EDTA buffer [40 mM Tris-acetate, 1 mM EDTA (pH 8.0)]. The gel was stained in ethidium bromide solution (0.5 µg/mL) for 30 min, and finally visualized on an ultra-violet trans-illuminator.

### Nuclease assay on single and double stranded DNA

UVI31+ (23 μM) was incubated with 300 ng each of linear pUC19 double stranded (ds) DNA and M13mp18 single stranded (ss) DNA for various times from 0–60 minutes in a buffer containing 50 mM sodium phosphate (pH 7.6) and 50 mM NaCl, 10 mM MgCl_2_ in a reaction volume of 20 μl at 37 °C. The reaction was stopped by adding 0.1% SDS and analyzed by electrophoresis in a 1% agarose gel containing 0.5 μg/ml EtBr. The images were analyzed by FIJI software and plotted as a function of reduction of ss- or ds-DNA band intensity with time and normalized to starting amounts of 100% of each substrate.

### Electrophoresis mobility shift assay (EMSA)

EMSA was carried out as described previously^72^. Briefly, 1 μM DNA (single stranded or double stranded) was incubated with varying concentrations of UVI31+ (0.5, 1, and 2 µM) in 10 mM sodium-phosphate buffer (pH 7.5) containing 10 mM NaCl and 1 mM MgCl_2_ in a total volume of 20 μL. The reactions were incubated for 5 min at 37 °C and were analyzed using 2% agarose gel electrophoresis by loading 10 μL from each reaction to the respective gel wells. Control reactions without any protein were also carried out for comparison. Quantification of the gel shift assay data was carried out using ImageJ^73^. Binding data were fitted to the equation (y = V_max_ * x/(K_d_ + x)) describing a sigmoidal curve^72^ and K_d_ values were calculated from the fit of the curve using Origin Pro 2018 software.

### Interaction of UVI31+ with DNA by Isothermal Titration Calorimetry (ITC)

We used ITC to characterize quantitatively the thermodynamics of UVI31+ binding to a self-complementary 12 mer ds-DNA. ITC experiments were performed using a VP-ITC Micro-Calorimeter (MicroCal Inc., Northampton, MA, USA) with UVI31+ (taken at a concentration of 75 μM) and self-complementary ds-(CGCGAATTCGCG) DNA taken at a concentration of 2 mM. The protein and the DNA were extensively dialyzed against similar buffer, containing 10 mM Tris–HCl (pH 7.5), 50 mM NaCl, 5% glycerol, 5 mM MgCl_2_, 0.1 mM EDTA, prior to the performance of experiments, to avoid heat signals that could arise while mixing nonequivalent buffers. All solutions were carefully degassed before each titration using equipment provided with the calorimeter. Each titration consisted of 5 μl injections of the self-complementary ds-(CGCGAATTCGCG) DNA into the 75 µM protein-containing sample cell (~1.5 ml) at 25 °C with a mixing speed of 220 r.p.m. Heats of dilution were determined by titrating the same ds-(CGCGAATTCGCG) DNA into the dialysis buffer or into the buffer containing the protein. The data were then integrated to generate curves in which the areas under the injection peaks were plotted against the concentration ratio of DNA to protein. Analysis of the data was performed using MicroCal ITC Origin software and isotherm were fitted with the sequential binding models.

## Electronic supplementary material

Supplementary Material
