# Supplementary material for: Structural characterization of a novel KH-domain containing plant chloroplast endonuclease
Source: Sci Rep. 2018 Sep 13;8:13750. doi: 10.1038/s41598-018-31142-w (PMC6137056; doi:10.1038/s41598-018-31142-w)
Supplement: Supplementary file 1 — Supplementary Material [file 41598_2018_31142_MOESM1_ESM.pdf]

# Supplementary Information

## Structural characterization of a novel KH-domain containing plant chloroplast endonuclease

Ashok K. Rout,[1, #] Himanshu Singh,[1,4,6 #] Sunita Patel,[4,5] Vandana Raghvan,[2] Saurabh Gautam,[7] R. Minda,[2] Basuthkar J. Rao,[2,8] Kandala V. R. Chary*[1,3,4]

Department of Chemical[1] and Biological[2] Sciences, Tata Institute of Fundamental Research, Mumbai, 400005, India
[3]Indian Institutes of Science Education and Research, Berhampur, 760010, Odisha, India
[4]Tata Institute of Fundamental Research, Center for Interdisciplinary Sciences, Hyderabad, 500075, India
[5]UM-DAE Centre for Excellence in Basic Sciences, Mumbai University Campus, Mumbai, India
[6]Department Chemistry and Pharmacy, Ludwig-Maximilians-University, Butenandtstr. 5-13, 81377, Munich, Germany
[7]Department of Chemistry, Indian Institute of Technology Delhi, Hauz Khas, New Delhi 110093, India

[8]Indian Institutes of Science Education and Research, Tirupati, 517501, Tirupati, India

# Ashok K Rout and Himanshu Singh share equal authorship.

*To whom correspondence should be addressed: Kandala V. R. Chary, Department of Chemical Sciences, Tata Institute of Fundamental Research, 1, Homi Bhabha Road, Colaba, Mumbai, 400005, India and Tata Institute of Fundamental Research, Center for Interdisciplinary Sciences, Hyderabad, 500075, India.
Tel.: +91 (22) 2278-2489; Fax: +91 (22) 2280-4610; Email: chary@tifr.res.in

**Scheme-S1:** Sequence alignment of UVI31+ and BolA

```
                       10        20        30        40        50        60
                        |         |         |         |         |         |
UVI31+        MRGSHHHHHHGSHVISSIASRGSMAEHQLGPIAGAIKSKVEAALSPTHFKLINDSHKHAG
BolA          ---------------GSSGSSG-MMIRER--IEEKLR----AAFQPVFLEVVDESYRHN-
                            .* .* * *  ::   *   ::    **:.*..::::::*::*

                       70        80        90       100       110       120
                        |         |         |         |         |         |
UVI31+        HYARDGSTASDAGETHFRLEVTSDAFKGLTLVKRHQLIYGLLSDEFKAGLHALSMTTKTP
BolA          --VPAGS------ESHFKVVLVSDRFTGERFLNRHRMIYSTLAEELSTTVHALALHTYTI
               .  **      *:**:: :.** *.*  :::**::**. *::*:.: :***:: * *

                     130
                      |
UVI31+       AEQ---------------
BolA         KEWEGLQDTVFASPPCRG
              *
```

**Scheme-S2:** Sequence alignment of the long-loop of UVI31+ and BolA

```
UVI31+       DSHKHAGHYARDGSTASDAGETH-
BolA         DES------YRHN---VPAGSESHF
             *.      * ::   .. ..*:*
```

**Table-S1:** Thermodynamic parameters for UVI31+ binding to ds- (CGCGAATTCGCG) DNA derived from ITC data.

| Sample | Macroscopic binding constant ($M^{-1}$) | Effective Apparent binding affinity $K_d$ (μM) | ΔH (kcal $mol^{-1}$) | ΔS (kcal $mol^{-1}$ $K^{-1}$) |
|---|---|---|---|---|
| UVI31+ | $K_1$=(3.05 ± 0.27)×$10^6$<br>$K_2$=(1.14 ± 0.08)×$10^4$ | 5.36 ± 0.3 | (-36.84 ± 0.23)<br>(-19.90 ± 0.80) | -0.094<br>-0.048 |

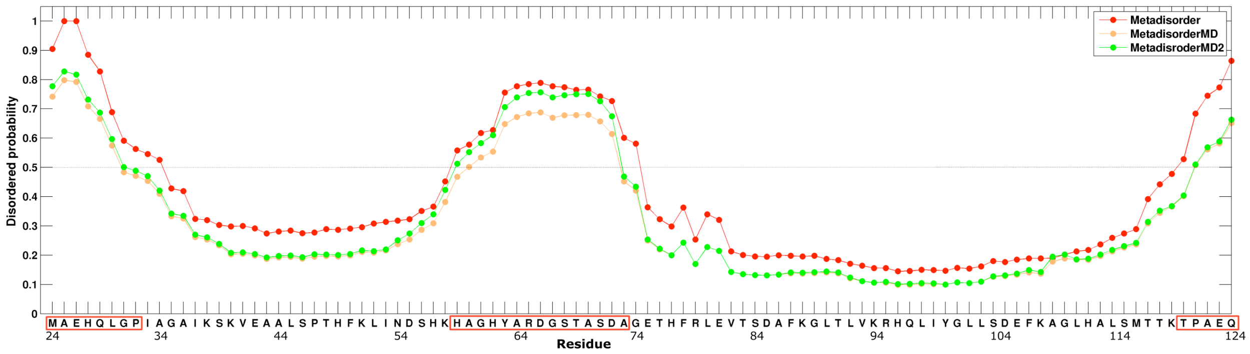


**Figure S1.** The disordered prediction probability of the UVI31+ sequence determined using Metadisorder web server. The probability value of a given residue greater than 0.5 is predicted to be in an intrinsically disorder state. The x-axis shows the residue names and the residue number at every 10th residue. The stretch of residues with disordered propensity are shown in red boxes on the x-axis.

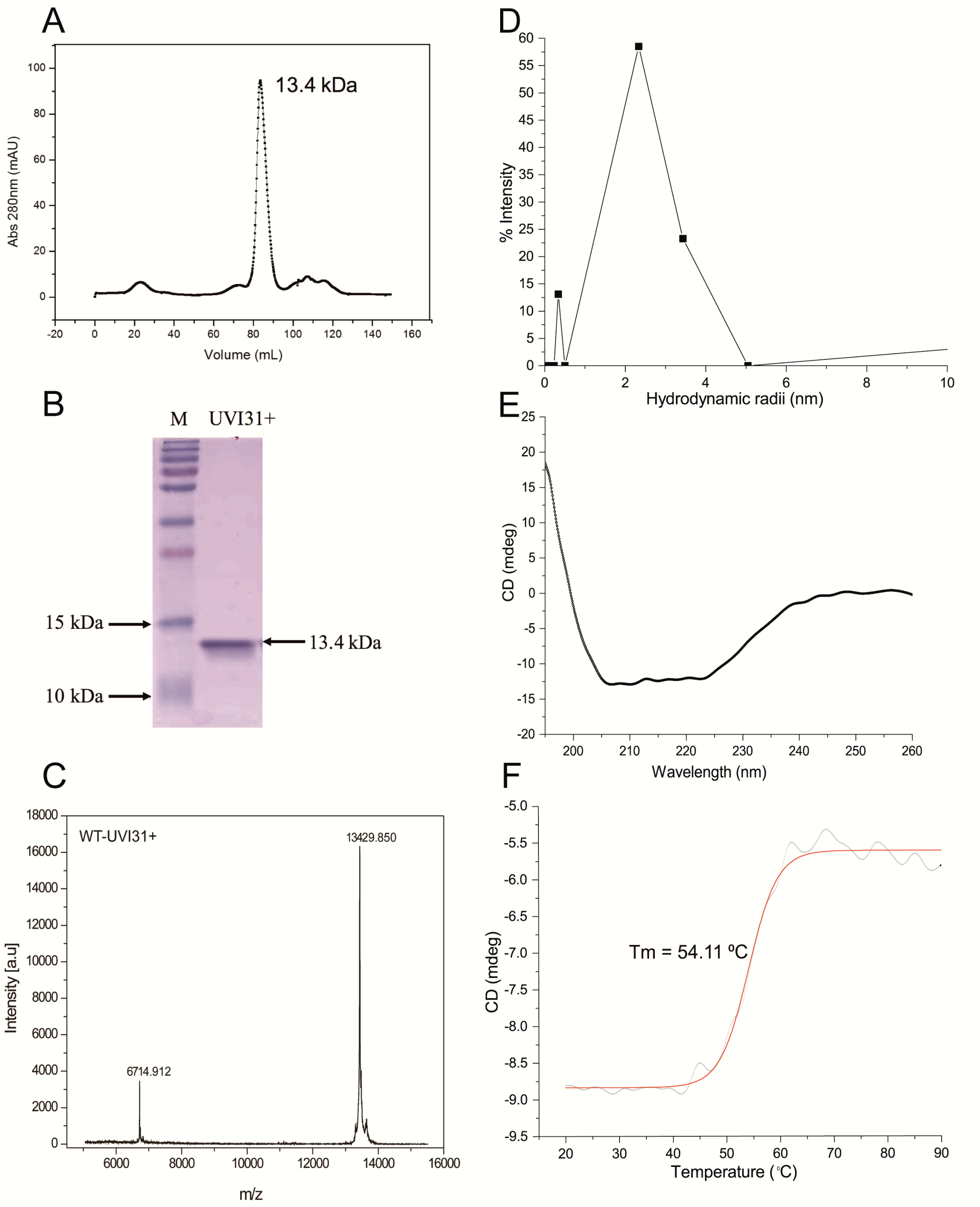


**Figure S2. (A)** Size-exclusion chromatography profile of UVI31+. **(B)** Purified UVI31+ band (right) shown on SDS-PAGE (15%) with marker (left). The gel image presented here is cropped for presentation point of view. **(C)** Matrix-assisted laser desorption ionization time-of-flight (MALDI-TOF) data of UVI31+ (M.W of 13429.85 Da) and expected molecular mass of UVI31+ from its sequence calculated to be 13300.93 Da. **(D)** Dynamics light scattering regularization plot of UVI31+. **(E)** Far-UV CD spectra of UVI31+. **(F)** Temperature dependence CD spectra of UVI31+.

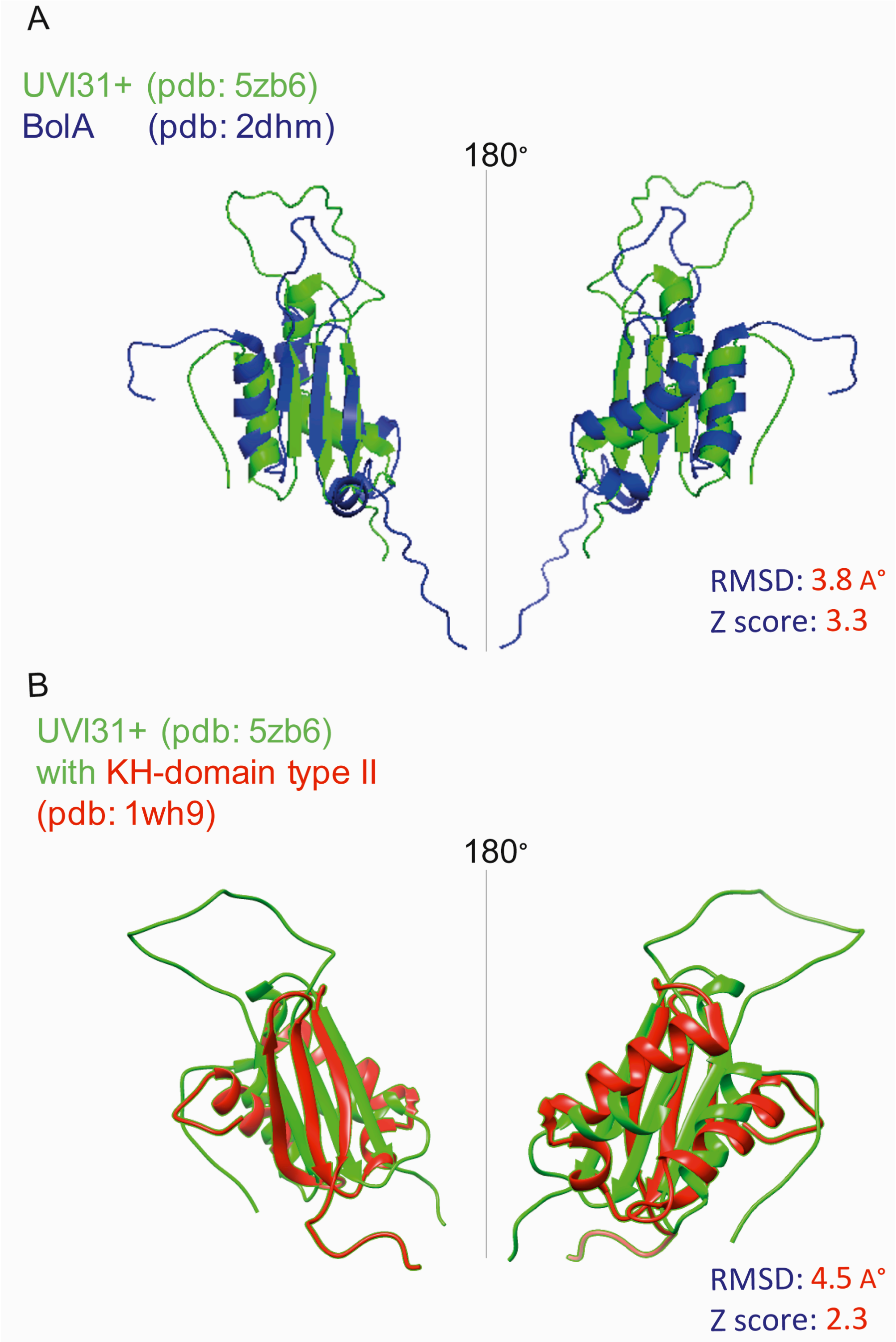


**Figure S3.** Structural homologues of UVI31+. **(A)** An overlay of the NMR derived structure of UVI31+ (Green) with BolA (left panel, Blue), Z-score = 3.3 and RMSD of 3.8 Å. **(B)** Type II KH-domain (Right panel, Red), Z-score = 2.3 and RMSD of 4.5 Å. The Z score were calculated from Dali web server for pairwise comparison of protein structures (http://ekhidna.biocenter.helsinki.fi/dali_lite/start).

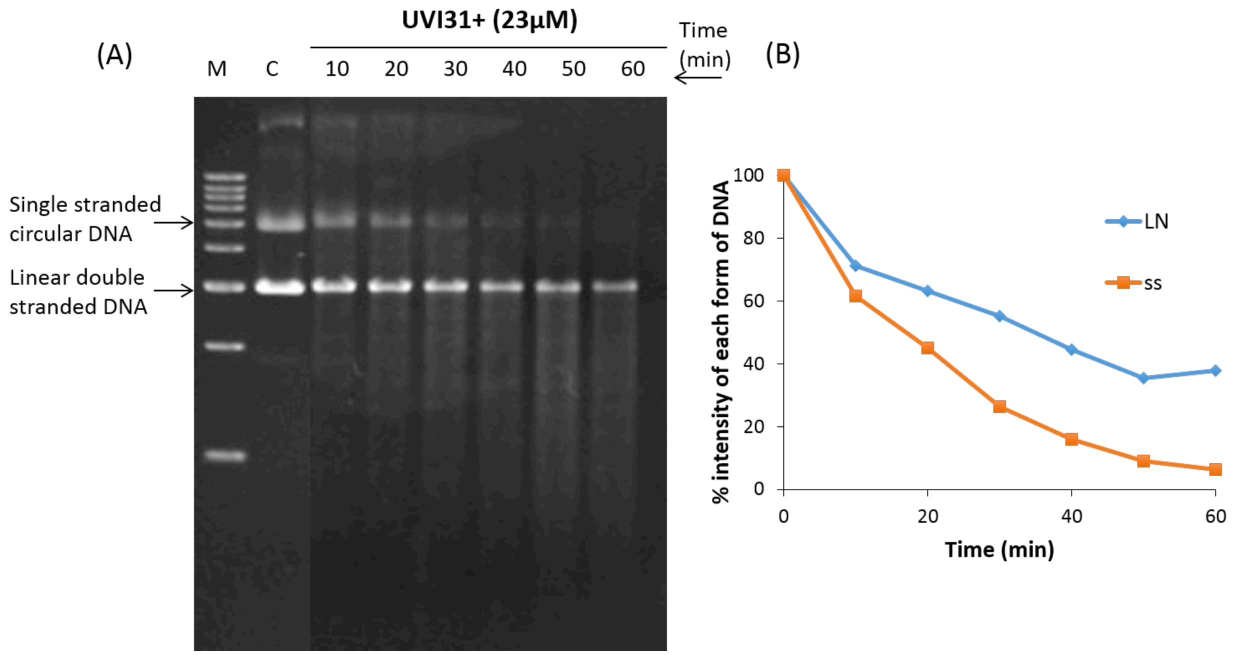


**Figure S4. (A)** Endonuclease activity on ss and ds DNA (A) UVI31+ (23 μM) activity on single stranded (ss) circular and linear double stranded (ds) DNA analyzed on a 1% agarose gel for 0-60 min. The image presented here is the full-length gel, cropped for presentation point of view. **(B)** Percentage band intensity reduction of single and linear double stranded DNA as a function of time.

A

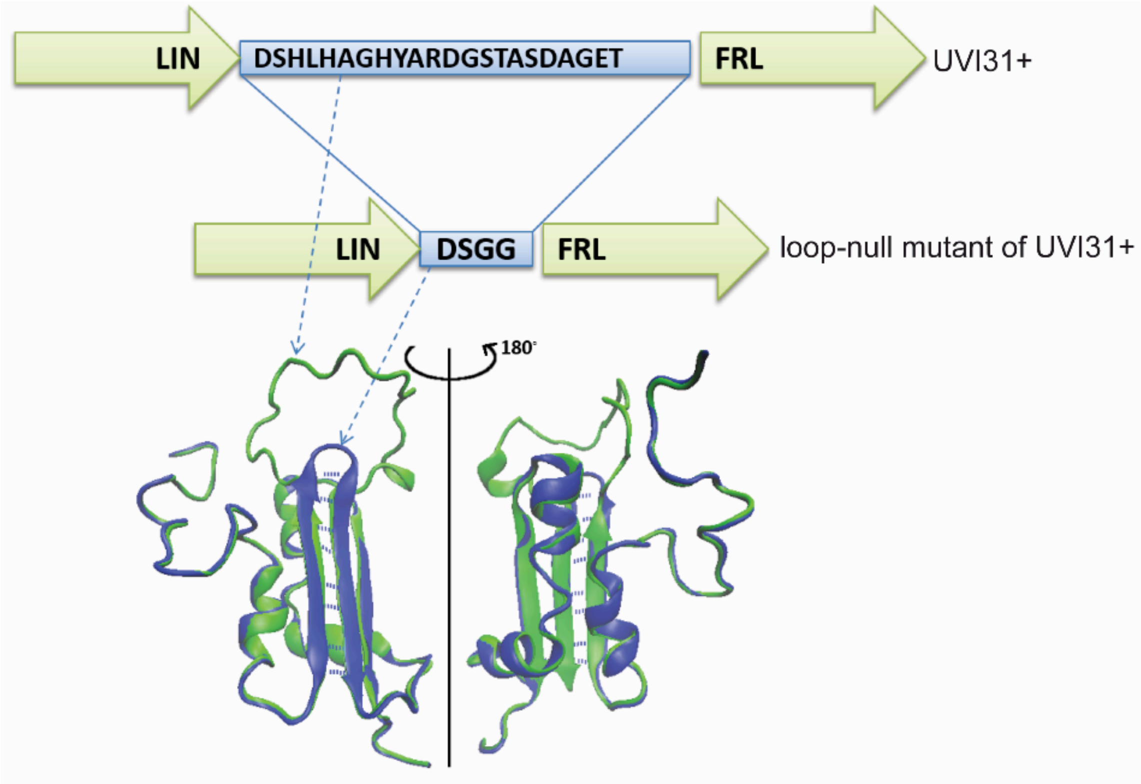


B

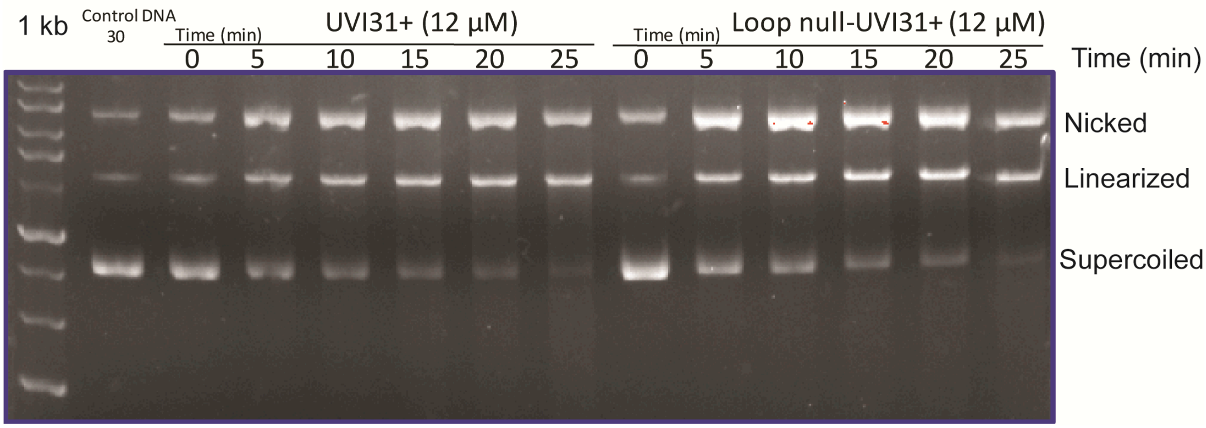


**Figure S5. (A)** Schematic representation of loop-null mutant of UVI31+ designed as described in Methods. **(B)** Endonuclease activity test of UVI31+ and loop-null mutant of UVI31+. The gel image presented here is the full-length gel, cropped for presentation point of view.

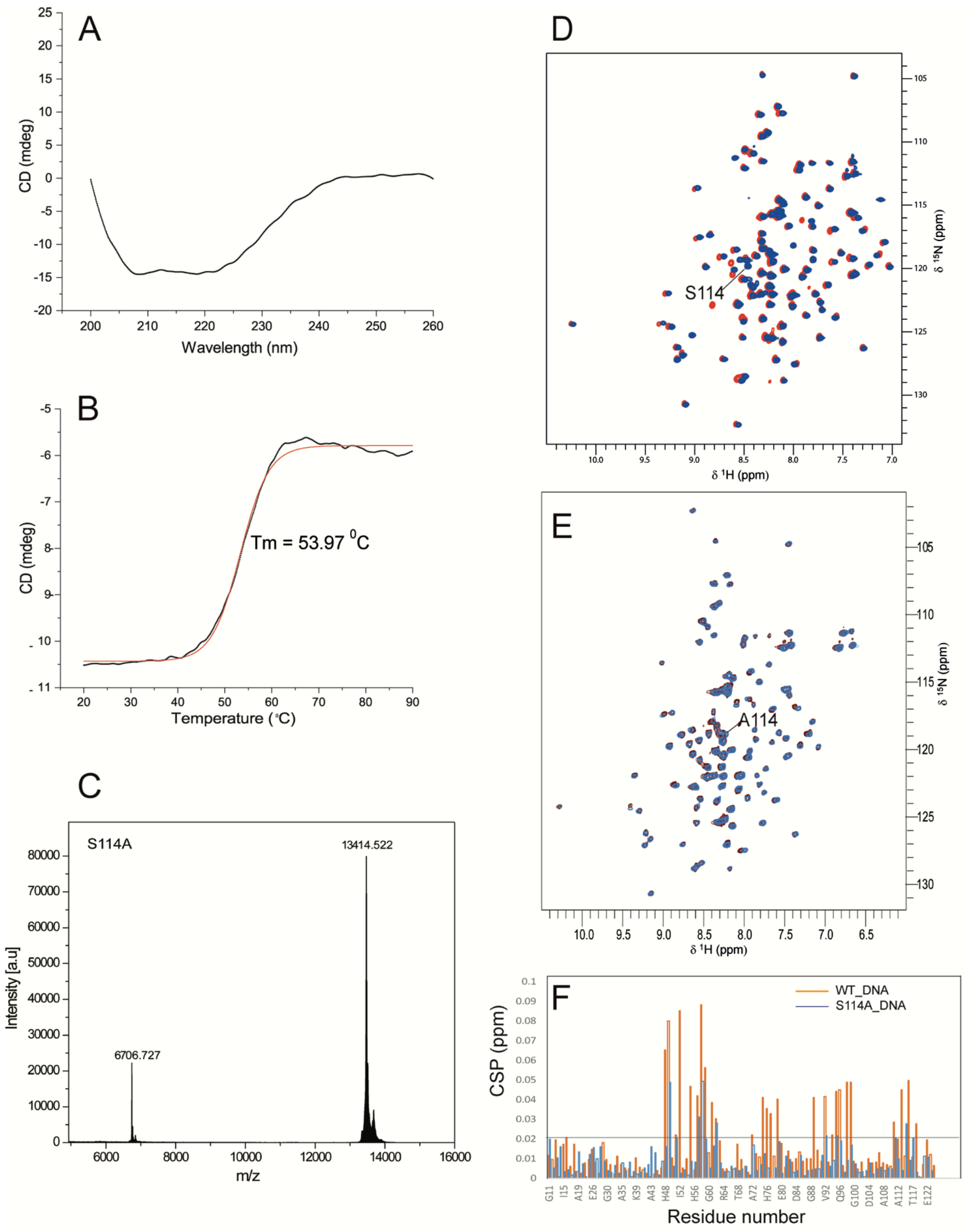


**Figure S6. (A)** Far-UV CD spectra of S114A-UVI31+ **(B)** Temperature dependence CD spectra of S114A-UVI31+ showing melting temperature ($T_m$) of 53.97 ºC. **(C)** Matrix-assisted laser desorption ionization time-of-flight (MALDI-TOF) data of S114A-UVI31+ (M.W of 13414.52 Da) and expected molecular mass of S114A mutant of UVI31+ from its sequence calculated to be 13284.93 Da. **(D)** Overlay of 2D [$^{15}$N, $^{1}$H]-so-fast-HMQC for UVI31+ (blue) and S114A-UVI31+ (red) **(E)** Overlay of 2D [$^{15}$N-$^{1}$H]-so-fast-HMQC for S114A-UVI31+ (sky blue) and S114A-UVI31+:DNA complex (brown). **(F)** CSP plot between WT with ds-DNA versus S114A with ds-DNA complex.

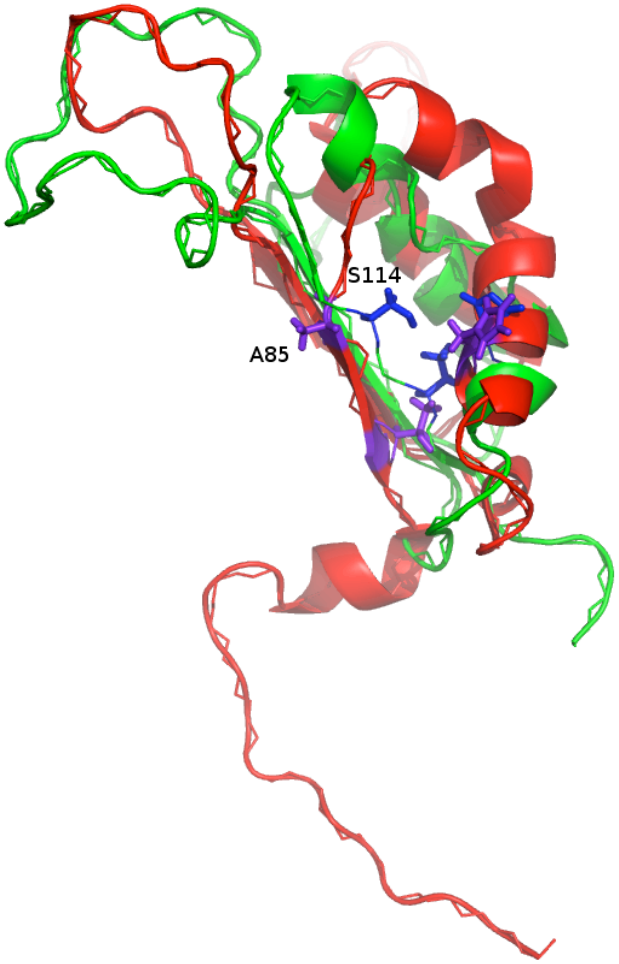


**Figure S7.** Structural alignment of UVI31+ (Green) (*pdb: 5ZB6*) and BolA (Red) (*pdb: 2DHM*), showing partial conservation of the catalytic triad.
